# Tracking of the origin of recurrent mutations of the *BRCA1* and *BRCA2* genes in the North-East of Italy and improved mutation analysis strategy

**DOI:** 10.1186/s12881-016-0274-6

**Published:** 2016-02-06

**Authors:** Giulia Cini, Massimo Mezzavilla, Lara Della Puppa, Elisa Cupelli, Alessio Fornasin, Angela Valentina D’Elia, Riccardo Dolcetti, Giuseppe Damante, Sara Bertok, Gianmaria Miolo, Roberta Maestro, Paolo de Paoli, Antonio Amoroso, Alessandra Viel

**Affiliations:** Experimental Oncology 1, CRO Aviano National Cancer Institute, Aviano, PN Italy; Institute for Maternal and Child Health, IRCCS “Burlo Garofalo”, Trieste, Italy; Institute of Hematology, Catholic University S. Cuore, Rome, Italy; Department of Economics and Statistics, University of Udine, Udine, Italy; Department of Medical and Biological Sciences, University of Udine, Udine, Italy; Cancer Bioimmunotherapy Unit, CRO Aviano National Cancer Institute, Aviano, PN Italy; Department of Endocrinology; Diabetes and Metabolic Disease, Division of Pediatrics, University Medical Centre Ljubljana, Ljubljana, Slovenia; Medical Oncology B, CRO Aviano National Cancer Institute, Aviano, PN Italy; Scientific Directorate; CRO National Cancer Institute, Aviano, PN Italy; Department of Medical Sciences, University of Torino, Torino, Italy; Division of Experimental Genetics, Sidra Medical and Research Center, Doha, Qatar

**Keywords:** *BRCA1*, *BRCA2*, Founder mutation, Breast cancer, SNaPshot®, Italian

## Abstract

**Background:**

About 20 % of hereditary breast cancers are caused by mutations in *BRCA1* and *BRCA2* genes. Since *BRCA1* and *BRCA2* mutations may be spread throughout the gene, genetic testing is usually performed by direct sequencing of entire coding regions. In some populations, especially if relatively isolated, a few number of recurrent mutations is reported, sometimes caused by founder effect.

**Methods:**

*BRCA1* and *BRCA*2 screening for mutations was carried out on 1114 breast and/or ovarian cancer patients complying with the eligibility criteria for BRCA testing. Haplotype analysis was performed on the probands carrying recurrent mutations and their relatives, using two sets of microsatellite markers covering the *BRCA1* (D17S588, D17S806, D17S902, D17S1325, D17S855, D17S1328, D17S800, and D17S250) and *BRCA2* (D13S220, D13S267, D13S171, D13S1701, D13S1698, D13S260, D13S290, D13S1246) *loci*. The DMLE + 2.2 software was used to estimate the age of *BRCA1* c.676delT and *BRCA2* c.7806-2A > G. A multiplex PCR and two different primer extension assays were optimized and used for genotyping the recurrent mutations of the two genes.

**Results:**

In the time frame of almost 20 years of genetic testing, we have found that five *BRCA1* and three *BRCA2* mutations are recurrent in a substantial subset of carriers from North-East Italy and neighboring Istria, where they represent more than 50 % of all mutations. Microsatellite analyses identified a common haplotype of different length for each mutation. Age estimation of *BRCA1* c.676delT and *BRCA2* c.7806-2A > G mutations revealed that they arose in the Friuli Venezia Giulia area about 86 and 94 generations ago, respectively. Suggestion of an association between *BRCA2* c.7806-2A > G and risk of breast cancer in males has emerged. Finally, we developed a simple and efficient pre-screeening test, performing an in-house primer extension SNaPshot® assay for the rapid identification of the eight recurrent mutations.

**Conclusions:**

Proofs of common ancestry has been obtained for the eight recurrent mutations. The observed genotype-phenotype correlation and the proposed rapid mutation detection strategy could improve the clinical management of breast and ovarian patients in North-East of Italy and neighboring geographic areas.

## Background

About 3-8 % of breast and ovarian cancers are hereditary and are due to constitutional mutations in cancer predisposing genes. Mutations of the *BRCA1* (OMIM 113705) and *BRCA2* (OMIM 600185) genes contribute to a significant number of hereditary cases and are inherited in a dominant autosomic manner with high penetrance [1].

Women carrying *BRCA1* mutations are particularly at risk of developing breast cancer at very early age and ovarian cancer during their life, while women carrying a *BRCA2* mutation tend to develop breast cancer later in their life and have a significantly lower susceptibility to ovarian cancer [2].

Thousands of different mutations have been found in both genes and are dispersed throughout the coding sequences, but the mutation spectra and proportion of high-risk mutated families varies widely among different populations. Some populations present a wide spectrum of different mutations, while particular ethnic groups present high frequency of a single or a few recurrent mutations, usually due to a founder effect [3, 4].

Among the several well established founder mutations, the 3 mutations of the Ashkenazi Jews (AJ), i.e. *BRCA1* c.68_69delAG and c.5266dupC, *BRCA2* c.5946delT, are worthy of particular mention because overall they account for 6.7-11.7 % of all breast cancer patients and 59 % of patients from high-risk breast cancer families in this population [3]. Among the approximate 30.000 entries of the BIC database [5], these 3 mutations are at the head of both “top 20 mutation frequencies lists”. However, this reflects in part their high recurrence also in non-Jews Caucasian populations, because these mutations likely existed before the Jewish diaspora.

Another famous and well-studied founder mutation is the *BRCA2* c.771del5, that is identifiable in approximately 8 % of both breast cancer and ovarian cancer Icelandic cases [6]. However, hundreds of recurrent and/or founder mutations have been reported in the last 15 years by several papers variably describing mutation types, frequency and distribution, haplotype sharing, common ancestor and mutation age, clinical phenotype and so on [3, 7, 8]. Several recurrent/founder mutations have been already reported also in Italy, each one confined within a limited regional geographic area. The most significant examples are *BRCA1* c.1378dupA and c.3228_3229delAG in Tuscany [9, 10], *BRCA1* c.4964del19 in Calabria and Sicily [11], *BRCA1* p.Val1688del in Veneto [12], *BRCA2* c.8537delAG and c.3723del3insAT in Sardinia [13, 14], and more recently, *BRCA1* p.Cys64Arg in the Lombardy region [15].

The present study focus on the *BRCA1* and *BRCA2* mutations that were observed multiple times among the patients of the North-East of Italy undergoing genetic testing for hereditary breast/ovarian cancer. Haplotype sharing and age calculation analyses are presented along with a multiplex genotyping test that has been developed for improving our screening strategy, allowing rapid identification of the patients carrying recurrent mutations.

## Methods

### Cases and controls

In the time frame of 19 years (1996–2014), 1114 breast and/or ovarian cancer patients complying with the eligibility criteria for *BRCA* testing [16], were screened for *BRCA1 or BRCA2* mutations. The updated criteria in use at the Centro di Riferimento Oncologico (CRO, National Cancer Institute, Aviano) were (a) three or more cases of breast and/or ovarian cancer at any age, with one case being a first-degree relative of the other two; (b) two first-degree relatives with breast cancer diagnosed before 50 years of age or at any age but with one case of bilateral breast cancer; (c) two first-degree relatives with ovarian cancer at any age or one ovarian cancer at any age and one breast cancer before the age of 50; and (d) one case of breast cancer before the age of 36 or breast cancer in male or breast and ovarian cancer in the same woman.

All patients were recruited in the setting of genetic counseling in Centers of the region Friuli Venezia Giulia (FVG), namely at CRO in Aviano (~65 %) and at the Institutes of Medical Genetics in Udine (~30 %) and Burlo Garofalo in Trieste (~5 %). Detailed family histories, including information on geographic origins, were obtained for all patients. Genealogic investigations did not reveal any relationship between individuals from different families. Informed consent for genetic testing and research was obtained from all participants. The genetic testing protocol and use of DNA samples for research purposes was evaluated and approved by the Local Independent Ethical Committee (CRO-15-1997).

Genomic DNA was purified from blood samples of each proband. In the majority of samples screening for mutations in the *BRCA1*/*BRCA2* genes was carried out by a combination of Denaturing High Performance Liquid Cromatography (DHPLC), direct DNA Sanger-sequencing and Multiplex-Ligation Dependent Probe Amplification (MLPA) techniques; Single Strand Conformation Polymorphism and Protein Truncation Test had been used instead of DHPLC and Sequencing for testing the first 300 cases, only.

Overall, the study was carried out on 62 apparently unrelated families carrying one of the 8 most recurrent mutations listed in Table 1 (39 *BRCA1*, 23 *BRCA2*). Besides the 62 mutated probands, a total of 120 relatives were also included in the study (54 carriers and 62 non-carriers).

**Table 1:** List of the most common *BRCA* mutations and their recurrence in the CRO Aviano database and BIC database

GENE	MUTATION^a^	BIC database	CRO database	CRO haplotype study
*BRCA1*	c.116G > A (p.Cys39Tyr)	5x	11x	7
*BRCA1*	c.181T > G (p.Cys61Gly)	239x	13x	7
*BRCA1*	c.676delT (p.Cys226Valfs*8)	16x	10x	9
*BRCA1*	c.1687C > T (p.Gln536*)	94x	12x	7
*BRCA1*	c.5266dupC (p.Gln1756Profs*74)	1088x	15x	9
*BRCA2*	c.5682C > G (p.Tyr1894*)	62x	7x	5
*BRCA2*	c.7806-2A > G (p.Ala2603_Arg2659del)	5x	19x	13
*BRCA2*	c.8878C > T (p.Gln2960*)	10x	6x	5

^a^The mutations are defined according to the Human Genome Variation Society guidelines; the symbol "*" indicates a predicted stop codon [46]

Ninety-one healthy blood donors, all born and resident in the North-Eastern Italy, were investigated to estimate allele frequencies and control haplotypes in the general population.

### Microsatellite analysis

**Fig. 1:**
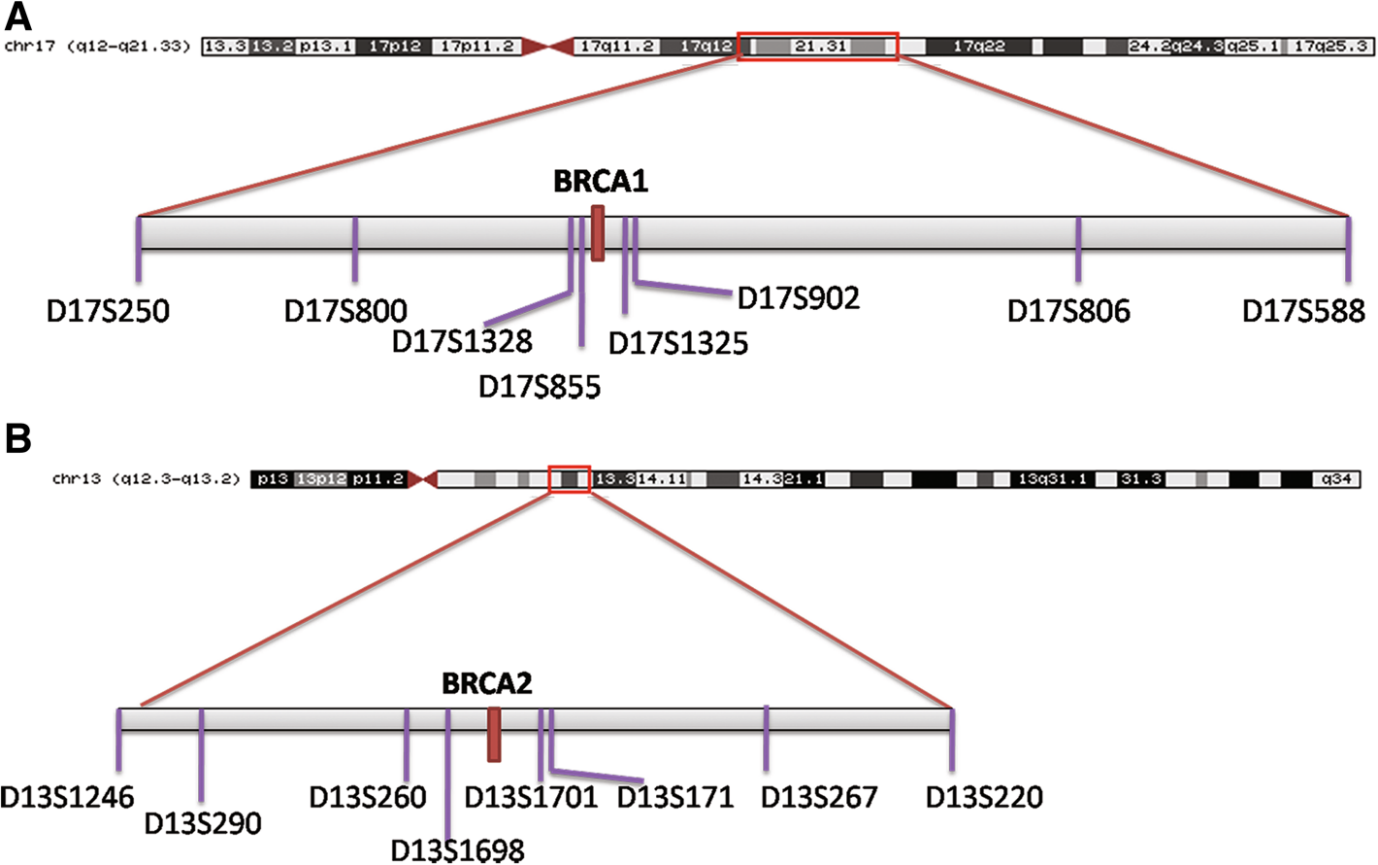
Schematic representation of (**a**) chromosome 17 and (**b**) chromosome 13. Relative positions of *BRCA1* and *BRCA2* genes and the flanking microsatellites are shown

Haplotype analysis was performed using two sets of 8 microsatellite markers covering the *BRCA1* and *BRCA2 loci* and spanning regions of approximately 11 Mb/8.7 cM and 4.1 Mb/7.0 cM, respectively. The following microsatellites, listed in order from telomere to centromere, were analyzed in *BRCA1*-mutated samples: D17S588, D17S806, D17S902, D17S1325, D17S855, D17S1328, D17S800, and D17S250. The microsatellites investigated in the *BRCA2*-mutated samples were: D13S220, D13S267, D13S171, D13S1701, D13S1698, D13S260, D13S290, D13S1246 (Fig. 1). PCR primer sequences were obtained from the Probe NCBI database [17] or designed using Primer Blast software [18]. Primer sequences and PCR conditions are available on request. PCR product size was evaluated by capillary electrophoresis on an ABI PRISM 3130 Sequencer using GeneMapper 4.0 software (Applied Biosystems/Life technologies, Foster City, CA, USA).

The distributions of allelic and haplotype frequencies in normal and mutated chromosomes were compared by Fisher’s exact tests; *P* < 0.05 and *P* < 0.01 were considered as a cut-off for statistical significance.

### Haplotyping and estimate of mutation age

Haplotypes were manually constructed for each mutation to minimize the number of recombinations. The cut off used for defining a founder effect was the association of the mutated *BRCA1* or *BRCA2* allele with a core haplotype spanning a minimum of 2 microsatellite markers.

The DMLE + 2.2 software developed by Reeve and Rannala [19] was used to estimate the age of *BRCA1* c.676delT and *BRCA2* c.7806-2A > G, as previously described [20, 21]. All the families with these two mutations clustered in the FVG.

The program, freely available online [22], uses a Bayesian approach to compare differences in linkage disequilibrium between the mutation and flanking markers in DNA samples from mutation carriers and controls. The software generates the marginal posterior probability density of mutation age based the following parameters: a) observed haplotypes or genotypes in normal and affected chromosomes; b) map distances between markers and mutation site; c) population growth rates and d) an estimated proportion of the mutation bearing chromosomes sampled.

Map distances were estimated on the basis of positions and physical distances given by the genetic map HapMap Phase II [23].

The population growth rate (^*gen*^*r*) was estimated as reported previously [20]. The total population of the FVG region currently comprises 1,229,363 people [24]. Historical and demographic data indicate that about 160,000 people lived in this area in year 1200 [25]. Accordingly, the average ^*gen*^*r* of this population was estimated to be 0.063 from 1200 to the present time, assuming 25 years/generation.

Taken for granted that the prevalence of *BRCA1* and *BRCA2* carriers is about 1:1000 each in the general population [26], three separate analyses were then performed, each using a different estimate for the proportion of sampled mutation-carrying chromosomes: 0.015, 0.01, and 0.005 [15].

### Primer extension SNaPshot® assay

Two different multiplex primer extension assays were optimized and used for genotyping the 8 recurrent mutations of the two genes. We used the SNaPshot® labeling chemistry (Applied Biosystem/Life technologies) that relies on single-base extension and termination using custom primers located upstream or downstream of the mutation site.

First of all, a single octaplex PCR was carried out for simultaneously amplify exons 3, 5, 20 and two portions of exon 11 of the *BRCA1* gene, plus exons 17, 22 and a portion of exon 11 of the *BRCA2* gene. The primers adopted for this test were the same we normally used for complete gene mutational screenings by DHPLC and/or direct sequencing (available upon request). Multiplex PCRs were performed with 0.1–0.4pmol of each primer and 2X QIAGEN Multiplex PCR Master Mix (Qiagen, Inc., Frederick, Maryland, USA) in a volume of 20 μl, according to manufacturer instructions and using the following conditions: denaturation at 95 °C for 15 min, followed by 40 cycles of 95 °C for 30 sec, 56 °C for 90 sec and 72 °C for 90 sec, and a final extension step of 72 °C for 10 min. When all expected PCR products and their sizes had been confirmed by electrophoresis on a 3 % agarose gel, the reaction was purified with ExoSAP (Exonuclease I and Shrimp Alkaline Phosphatase, GE-Healthcare, Buckinghamshire, UK) 15 min at 37 °C followed by 15 min at 75 °C) to remove excess dNTP and primers.

Multiplex nucleotide primer extension was carried out in a final volume of 10 μl containing 3 μl of purified PCR product, 0.2pM of each internal primer, 1 μl of 5X Sequencing Buffer (Applied Biosystem/Life technologies), and 2.5 μl of SNaPshot® MultiplexReady Reaction Mix (Applied Biosystems/Life technologies). Internal primers were constructed to have Tm of approximately 60 °C and sizes between 20 and 27 nucleotides, but with added poly(A) tails of different lengths to their 5' end (Table 2). The *BRCA1* primer pool comprised 5 primers, while the *BRCA2* primer pool included 3 primers.

**Table 2:** Internal SNaPshot® primers for the analysis of 8 *BRCA* recurrent mutations

GENE	Mutation	Primer name	Sequence	Orientation^a^	Size	ddNTP wt/mut	Signal colour
*BRCA1*	c.116G > A	1snap116-S	AAAAAAAAACAAGGAACCTGTCTCCACAAAGT	S	32	G/A	Blue/Green
*BRCA1*	c.181T > G	1snap181-S	AACAGAAGAAAGGGCCTTCACAG	S	23	T/G	Red/Blue
*BRCA1*	c.676delT	1snap676-AS	AAAAAAAAAGTTACATCCGTCTCAGAAAATTCACA	AS	35	A/G	Green/Blue
*BRCA1*	c.1687C > T	1snap1687-AS	AAAAAAAAAAAAACTATTGGGTTAGGATTTTTCTCATTCT	AS	40	G/A	Blue/Green
*BRCA1*	c.5266dupC	1snap5266-S	AAAGCGAGCAAGAGAATCCC	S	20	A/C	Green/Black
*BRCA2*	c.5682C > G	2snap5682-S	AAAAAAAACGAAAATTATGGCAGGTTGTTA	S	30	C/G	Black/Blue
*BRCA2*	c.7806-2A > G	2snap7806-AS	TGGAGTGTCACACAGAGCCC	AS	20	T/C	Red/Black
*BRCA2*	c.8878C > T	2snap8878-AS	GTTGTGACATCCCTTGATAAACCTT	AS	25	G/A	Blue/Green

^a^ *S* sense, *AS* antisense

The reaction was performed as recommended by the manufacturer in a thermal cycler (25 cycles), then treated by SAP (GE-Healthcare) 60 min at 37 °C and 15 min at 75 °C, run on the ABI PRISM 3130 Genetic Analyzer and evaluated with GeneMapper software (Applied Biosystems/Life technologies).

## Results

### Frequency of *BRCA* recurrent mutations

Overall, following the mutational screening of 1114 eligible probands, different *BRCA1*/*BRCA2* deleterious mutations were identified in 221 unrelated patients (18.9 %). Thirty-five *BRCA1* and 26 *BRCA2* mutations were unique, while 15 *BRCA1* and 19 *BRCA2* mutations were recurrent in 2–18 families. On the whole, 160 out of 1114 probands had a recurrent mutation.

**Fig. 2:**
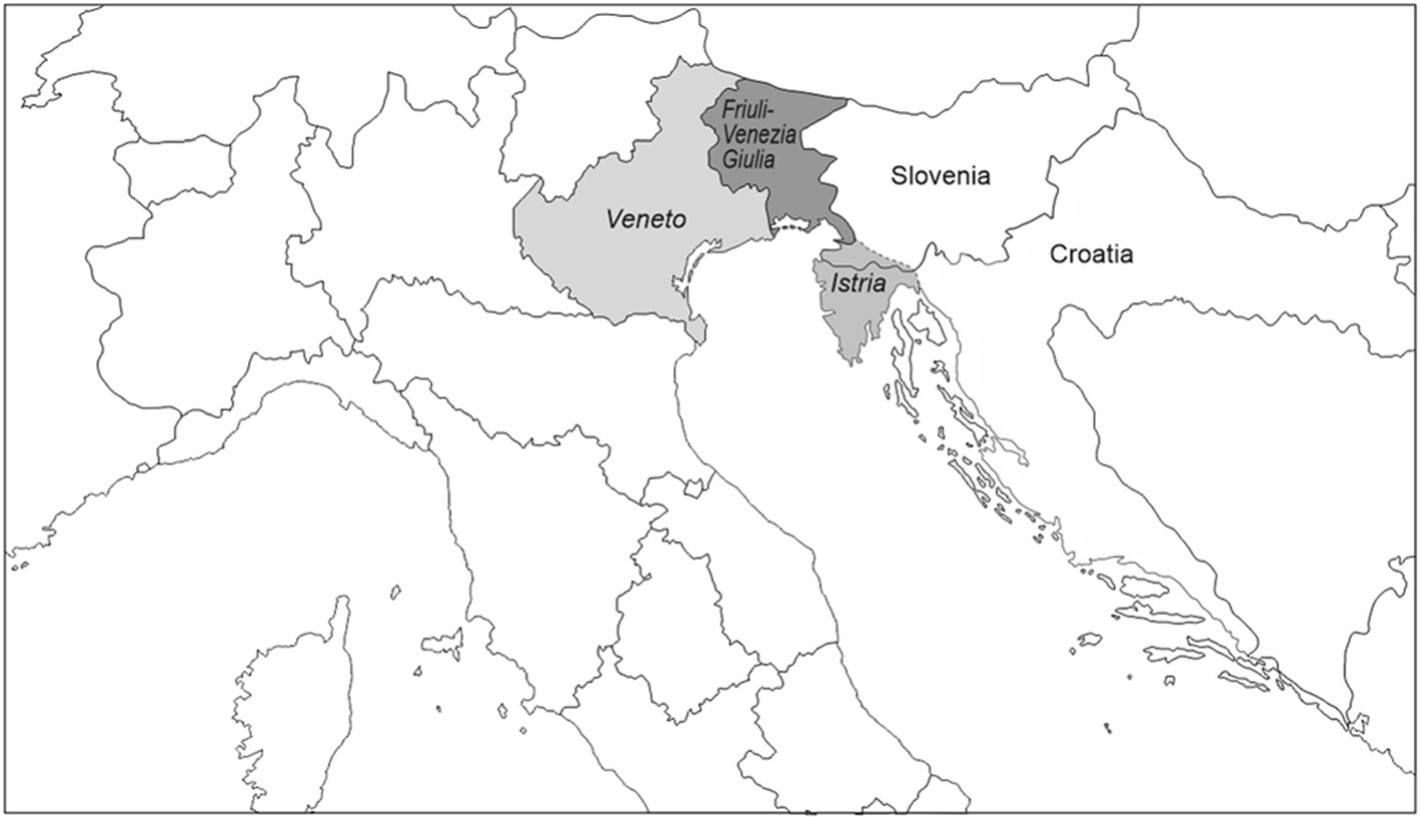
Map of North-East Italy and Istria. In grey, Italian official regions FVG and Veneto, and geographical region of Istria (Italy, Slovenia and Croatia)

We focused our attention to the 8 sequence variants listed in Table 1, which had a recurrence of at least 6 times. Overall, these mutations were responsible for the increased genetic risk in 93 unrelated probands/families, which represented 42 % (93/221) of the total number of cases with identified *BRCA* deleterious mutations in our Center. One hundred and fourty-seven of 221 mutated probands were born and resident in North-Eastern Italy, specifically in different provinces of the FVG and Veneto regions, or came from the neighboring Istria, a peninsula previously Italian but split between Italy, Croatia and Slovenia after the second world war (Fig. 2). Among this subgroup, 80 families carried the 8 common variants. Therefore, by restricting the evaluation to the patients sharing this common geographic origin, the frequency of the 8 recurrent mutations increased to 54 % (80/147).

### Haplotype analysis and age estimation

To investigate a possible founder effect, allele and haplotype analyses were performed on 62 families carrying one of the 8 recurrent *BRCA* mutations. For this analysis only families enrolled within the end of year 2011 were selected (Table 1). In details, 100 individuals (39 probands and 61 relatives) from 39 families segregating 5 recurrent *BRCA1* mutations, 78 individuals (23 probands and 55 relatives) from 23 families segregating 3 recurrent *BRCA2* mutations and the 91 control subjects were investigated.

The D17S1325 marker was not informative and was then excluded from the analysis.

The most common allele among the probands was considered for each microsatellite marker flanking the *BRCA1* and *BRCA2 loci*, and its frequency was compared between cases and controls. Statistically significant differences in allele frequencies between mutated probands and normal controls were observed for the markers located closer to each *BRCA1* and *BRCA2* variant (Tables 3, 4). In particular, D17S902 and D17S855 showed significant differences for all five *BRCA1* mutations, and D13S1698 for all three *BRCA2* mutations (*p* < 0.05).

**Table 3:** Frequencies of the most common microsatellite alleles of the *BRCA1* region in mutated probands and controls

		Markers^a^						
		*D17S588*	*D17S806*	*D17S902*	*D17S855*	*D17S1328*	*D17S800*	*D17S250*
		48215496	45811789	41647103	41204744	41159779	39056258	37152091
		136–160	136–182	137–173	143–157	247–267	166–178	*145–163*
c.116G > A	*Alleles* ^b^	156	170	149	149	247	170	149
	Cases (14)^c^	4 (0.29)	6 (0.43)	6 (0.43)	8 (0.57)	11 (0.79)	5 (0.36)	5 (0.36)
	Controls (140–180)^c^	52 (0.37)	30 (0.17)	32 (0.18)	28 (0.16)	146 (0.81)	80 (0.45)	45 (0.26)
	*P* value^d^	n.s.	<0.05	<0.05	<0.01	n.s.	n.s.	n.s.
c.181T > G	*Alleles* ^b^	148;156^e^	174	141	153	247	166	147
	Cases (14)^c^	4 (0.29);4 (0.29)	3 (0.21)	10 (0.71)	9 (0.64)	14 (1.0)	6 (0.43)	4 (0.29)
	Controls (140–180)^c^	33 (0.24);52 (0.37)	16 (0.09)	24(0.13)	24 (0.13)	146 (0.81)	16 (0.09)	23 (0.13)
	*P* value^d^	n.s.	n.s.	<0.01	<0.01	n.s.	<0.01	n.s.
c.676delT	*Alleles* ^b^	154	170	149	149	247	174	149
	Cases (18)^c^	10 (0.56)	10 (0.56)	10 (0.56)	9 (0.50)	16 (0.89)	10 (0.56)	5 (0.36)
	Controls (140–180)^c^	44 (0.31)	30 (0.17)	32 (0.18)	28 (0.16)	146 (0.81)	38 (0.21)	45 (0.26)
	*P* value^d^	n.s.	<0.01	<0.01	<0.01	n.s.	<0.01	n.s.
c.1687C > T	*Alleles* ^b^	152	170	145	145	247	170	149
	Cases (14)^c^	5 (0.36)	7 (0.50)	8 (0.57)	7 (0.50)	12 (0.86)	9 (0.64)	4 (0.29)
	Controls (140–180)^c^	20 (0.14)	30 (0.17)	19 (0.11)	39 (0.22)	146 (0.81)	80 (0.45)	24 (0.14)
	*P* value^d^	n.s.	<0.01	<0.01	<0.05	n.s.	n.s.	n.s.
c.5266dupC	*Alleles* ^b^	154	170	141	151	247	170	153
	Cases (18)^c^	5 (0.28)	7 (0.39)	6 (0.33)	12 (0.67)	17 (0.94)	9 (0.50)	3 (0.17)
	Controls (140–180)^c^	44 (0.31)	30 (0.17)	24 (0.13)	42 (0.24)	146 (0.81)	80 (0.45)	24 (0.14)
	*P* value^d^	n.s.	n.s.	<0.05	<0.01	n.s.	n.s.	n.s.

^a^Physical map positions on chromosome 17 based on Genome Reference Consortium Human Build 37 (GRCh37) [47] and allele size ranges are indicated for each *locus*

^b^The most frequent allele of the cases is reported for each *locus*. Alleles showing statistically significant association (corrected *P* value < 0.05) are underlined

^c^Total number of case and control chromosomes in parentheses. Number of chromosomes carrying the indicated alleles are reported for each *locus*, along with the allele frequency in parentheses

^d^ *n.s*. not significant

^e^Two common alleles with similar frequency were observed at this *locus*

**Table 4:** Frequencies of the most common microsatellite alleles of the *BRCA2* region in mutated probands and controls

		Markers^a^							
		*D13S220*	*D13S267*	*D13S171*	*D13S1701*	*D13S1698*	*D13S260*	*D13S290*	*D13S1246*
		35169554	34264119	33253912	33144530	32704522	32436758	31429174	31105437
		187–203	144–160	226–242	283–311	151–179	154–172	174–192	191–211
c.5682C > G	*Alleles* ^b^	189	156	226	299	155	162	174	201
	Cases (10)^c^	5 (0.5)	3 (0.3)	5 (0.5)	6 (0.6)	6 (0.6)	5 (0.5)	7 (0.7)	4 (0.4)
	Controls (142–182)^c^	59 (0.42)	38 (0.22)	40 (0.22)	51 (0.29)	46 (0.25)	19 (0.11)	105 (0.6)	26 (0.14)
	*P* value^d^	n.s.	n.s.	n.s.	n.s.	<0.05	<0.01	n.s.	n.s.
c.7806-2A > G	*Alleles* ^b^	199	144	230	299	155	160	174	205
	Cases (26)^c^	10 (0.38)	19 (0.73)	18 (0.69)	16 (0.62)	16 (0.62)	14 (0.54)	20 (0.77)	9 (0.35)
	Controls (142–182)^c^	34 (0.24)	68 (0.40)	73 (0.41)	51 (0.29)	46 (0.25)	28 (0.16)	105 (0.6)	60 (0.33)
	P value^d^	n.s.	<0.01	<0.02	<0.01	<0.01	<0.01	n.s.	n.s.
c.8878C > T	*Alleles* ^b^	189	144	240	287	173	168	174	199
	Cases (10)^c^	4 (0.4)	5 (0.5)	7 (0.7)	5 (0.5)	5 (0.5)	4 (0.4)	8 (0.8)	4 (0.4)
	Controls (142–182)^c^	34 (0.24)	68 (0.4)	61 (0.34)	22 (0.12)	4 (0.02)	28 (0.16)	105 (0.6)	32 (0.18)
	*P* value^d^	n.s.	n.s.	<0.05	<0.01	<0.01	n.s.	n.s.	n.s.

^a^Physical map positions on chromosome 13 based on Genome Reference Consortium Human Build 37 (GRCh37) [47] and allele size ranges are indicated for each *locus*

^b^The most frequent allele of the cases is reported for each *locus*. Alleles showing statistically significant association (corrected *P* value < 0.05) are underlined

^c^Total number of case and control chromosomes in parentheses. Number of chromosomes carrying the indicated alleles are reported for each *locus*, along with the allele frequency in parentheses

^d^ *n.s*. not significant

**Fig. 3:**
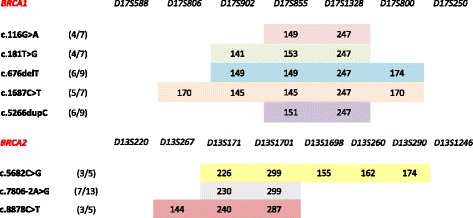
Shared haplotype of common mutations: The minimum haplotypes and the size of the microsatellite markers associated with each mutation are shown. These core haplotypes were found in at least 50 % of the mutated chromosomes, but were absent or rare in the 182 control chromosomes (*p* < 0.05 in all cases). For each mutation, number of chromosomes with the core haplotype out of the total number of mutated chromosomes are indicated in brackets

For the haplotype analysis, evaluation of the informative microsatellites was performed on probands and, when possible, on additional family members. Sharing of common haplotypes of different length was evident, suggesting a founder effect for all examined mutations. Indeed, core haplotypes extending over 2 to 5 markers were associated with each mutation, since they were present in at least 50 % of mutated chromosomes, but were absent or rare in the control chromosomes (Fig. 3).

The *BRCA1* c.676delT and the *BRCA2* c.7806-2A > G were further investigated. All the 7 informative families with c.676delT shared a common haplotype at *loci* D17S902, D17S855 and D17S1328 (149-149-247) spanning a region of approximately 487 kb (0.14 cM) [23] (Table 5). The same haplotype was compatible with the observed genotypes of two additional single individuals for which the phase could not be explored, due to the lack of additional family members. Furthermore, this common haplotype was not found in 94 % of control chromosomes, but could not be excluded in the remaining 6 % (data not shown).

**Table 5:** Haplotype analyses in the *BRCA1* c.676delT carrier families

	*D17S588*	*D17S806*	*D17S902*	*D17S855 (BRCA1)* ^*a*^	*D17S1328*	*D17S800*	*D17S250*
BR128	**154**-156	168-**170**	**149**-149	**149**–153	**247**–247	170–**174**	153–**159**
BR328	**154**–154	170–172	149–153	147–149	**247**–247	166–174	149–159
BR384	148–**154**	**170**–178	145–**149**	**149**–155	**247**–249	**174**–174	149–**159**
BR392	**154**–159	**168**–170	**149**–153	145–**149**	**247**–247	**174**–174	145–**155**
BR573	154–**156**	**162**–176	**149**–153	147–**149**	**247**–251	170–**174**	149–**153**
BR613	138–**154**	**170**–172	**149**–153	143–**149**	**247**–247	170–**174**	**149**–149
BR704	**140**–154	**170**–176	145–**149**	147–**149**	**247**–247	170–176	145–**155**
BR977	**154**–156	**170**–174	**149**–157	**149**–153	**247**–247	170–**174**	**151**–153
BR1091	148–154	**170**–170	149–157	149–151	**247**–247	170–174	151–161

Alleles segregating with the mutation inside each family are represented in bold type. The 149–149–247 shared haplotype is underlined

^a^ D17S855 is located into the *BRCA1 locus*

Six informative families with c.7806-2A > G shared an identical haplotype at *loci* D13S267, D13S171 and D13S1701 (144–230–299) spanning a region of approximately 1120 kb (1.58 cM) [23] (Table 6). The same haplotype combination was still likely in the 7 remaining families/individuals. Conversely, this haplotype could be excluded in 88 % of the tested controls (data not shown). In addition, segregation analysis of the nearest c.7806-14 C/T polymorphism (rs9534262) demonstrated that all 11 informative mutant alleles also shared nucleotide T at this position (Table 6), despite its reported population frequency of 0.453 [27].

**Table 6:** Haplotype analyses in the *BRCA2* c.7806-2A > G carrier families

	*D13S220*	*D13S267*	*D13S171*	*D13S1701*	rs9534262 (*BRCA2)*	*D13S1698*	*D13S260*	*D13S290*	*D13S1246*
BR6	**189**-193	**144**-144	**230**-240	287-**299**	C/**T**	**155**-159	158-**160**	**174**-174	**203**-203
BR60	189-**199**	**144**-144	**230**-230	**299**-299	T/**T**	**155**-155	158-**160**	**174**-174	**203**-205
BR85	**199**-199	**144**-144	230-240	287-299	T/**T**	**159**-159	**158**-158	**174**-174	**203**-203
BR195	189-**199**	**144**-144	**230**-240	287-**299**	T/**T**	155-159	160-162	**174**-174	203-**205**
BR243	**189**-189	**144**-156	**230**-230	295-**299**	T/**T**	**155**-171	**160**-160	**174**-188	**205**-205
BR312	189-199	144-150	230-240	287-299	C/T	155-159	160-166	**174**-174	201-205
BR434	189-195	144-156	**230**-240	295-**299**	T/**T**	**155**-155	**160**-162	174-188	201-**203**
BR594	189-195	144-150	230-230	295-299	T/**T**	**155**-155	156-160	174-188	205-208
BR608	193-**195**	**144**-144	**230**-230	**299**-307	C/**T**	**155**-163	**160**-170	**174**-188	201-**205**
BR953	189-**199**	**144**-152	**230**-230	**299**-299	T/**T**	**155**-159	**160**-168	**174**-174	**205**-205
BR1009	193-199	**144**-144	226-230	287-299	C/T	155-159	160-168	174-188	201-211
BR1013	189-199	144-156	226-230	**299**-299	T/**T**	155-159	**160**-160	**174**-174	201-207
CFS864	**199**-199	**144**-156	**230**-240	295-299	C/**T**	**155**-155	**160**-168	**174**-176	199-**207**

Alleles segregating with the mutation inside each family are represented in bold type. The 144-230-299 shared haplotype is underlined

All 22 families with these two latter mutations clustered in the FVG region (provinces of Pordenone, Udine, Trieste and Gorizia).

**Fig. 4:**
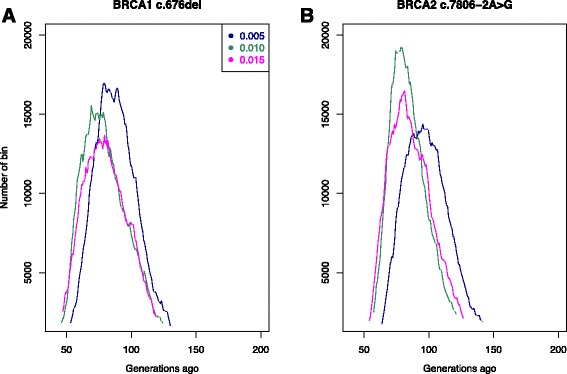
Results from DMLE analyses. Posterior probability density of the mutation age for different proportions of mutation-carrying chromosomes sampled: 0.005, 0.01, 0.015 (blue, green, magenta, respectively). **a**: Estimates for *BRCA1* c.676del. **b**: Estimates for *BRCA2* c.7806 − 2A > G

Given the three different proportions of sampled mutation-carrying chromosomes (0.005, 0.01, 0.015) the mutation age estimates for *BRCA1* c.676delT were approximately 91 generations (standard deviation 22, 95 % 46–126), 85 generations (standard deviation 22, 95 % 43–120), 83 generations (standard deviation 21, 95 % 40–116) (Fig. 4a). Considering a generation time of 25 years the corresponding ages in years were 2275, 2125, 2075. For *BRCA2* c.7806-2A > G the mutation ages were approximately 102 generations (standard deviation 22, 95 % 68–137), 89 generations (standard deviation 18, 95 % 61–117), 90 generations (standard deviation 21, 95 % 59–120) (Fig. 4b). The age estimates are 2550 years, 2225 years, and 2250 years.

### Genotype-phenotype correlations

Data on sex, tumor, age and family history of all the cases of our database with the *BRCA1* c.676delT and *BRCA2* c.7806-2A > G are summarized in Table 7. Ovarian cancer was present in 6/10 families with the *BRCA1* c.676delT (1 to 5 patients per family) and in 4/18 families with the *BRCA2* c.7806-2A > G (1 to 3 patients per family). Male breast cancer was absent in the *BRCA1* c.676delT pedigrees, but was recorded for the *BRCA2* c.7806-2A > G in 7 out 18 (39 %) families. On the contrary, male breast cancer was observed in 13 out 80 families (16 %) carrying c.5682C > G and c.8878C > T or other *BRCA2* mutations of our dataset (*p* = 0.04907). It is worth of noting that there were 3 males affected by breast cancer in one family with the c.7806-2A > G mutation (Table 7).

**Table 7:** Clinical phenotype of families with founder mutations

	Proband			Family		
	Sex^a^	BC^b^age	OC^b^age	F-BC^c^	OC^c^	M-BC^c^
*BRCA1*c.676delT						
BR128	F	41, 51	-	3 (2)	0	0
BR328	F	23	–	3 (1)	1	0
BR384	F	50, 56	–	3 (1)	1	0
BR392	F	30	–	4 (1)	0	0
BR573	F	–	52	1	3 (2)	0
BR613	F	–	53	4 (1)	5 (2)	0
BR704	F	29, 31, 31	–	1 (1)	1	0
BR977	F	58	–	4 (1)	0	0
BR1091	F	23, 29	–	2 (1)	0	0
BR1450	F	42	–	2(1)	2	0
*BRCA2* c.7806–2A > G						
BR6	F	60	–	5 (1)	0	1(1)
BR60	F	45	–	8 (3)	2 (2)	0
BR85	F	41	–	3 (1)	0	0
BR195	M	50	–	5	0	3 (1)
BR243	M	70	–	4	0	1 (1)
BR312	F	47	–	2 (1)	0	1
BR434	F	36	–	4 (3)	3	0
BR594	F	36	–	1 (1)	1	0
BR608	M	67	–	3 (2)	0	1 (1)
BR953	F	39	–	4 (3)	0	1
BR1009	F	36	–	3 (1)	0	0
BR1013^d^	F	–	–	3	0	0
CFS864	F	–	59	1	1 (1)	0
BR1214	F	38	–	7	0	0
BR1341	F	76	–	2	0	0
BR1267	F	45	–	1bil	0	0
BR1548	F	48	–	5	0	0
BR1481	F	48, 69	61	1	0	2

^a^ *F* female, *M* male

^b^ Years at diagnosis of tumors in probands, *BC* breast cancer, *OC* ovarian cancer

^c^Number of affected cases, including proband; in parentheses the number of carriers, ascertained or inferred, *F*–*BC* female breast cancer, *M–BC* male breast cancer

^d^Healthy young proband, no affected relatives were alive and available for gene testing

### SNaPshot® genotyping strategy

**Fig. 5:**
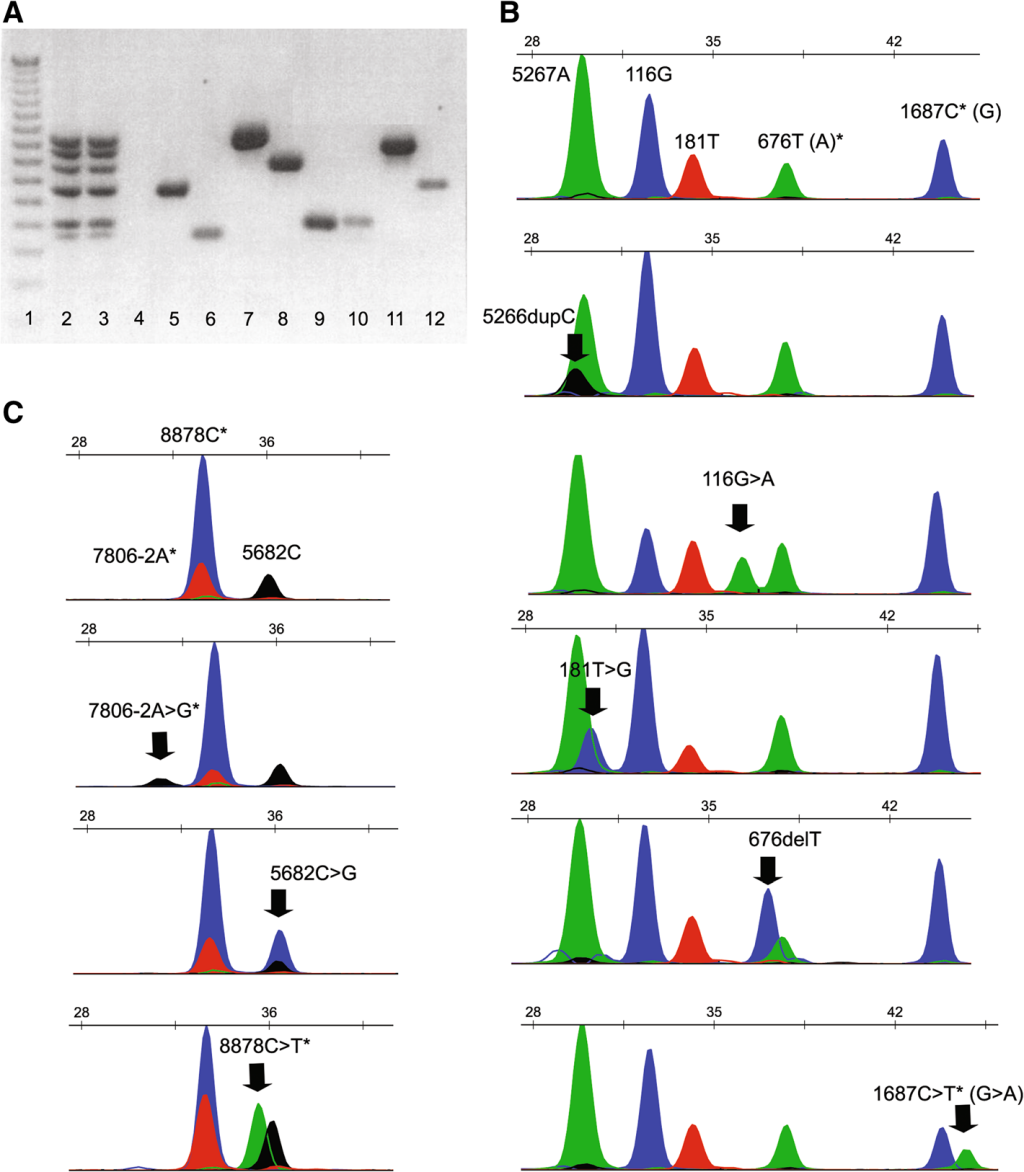
SNaPshot® analyses. **a**: Example of the multiplex and single amplified products; lane 1, 50 bp marker; lane 2 and 3, multiplex PCR of two different samples; lane 4, negative control of multiplex PCR; lane 5–12: single PCR products, in the order *BRCA1* exon 3, 5, 11–1, 11–3, 20 and *BRCA2* exon 17, 11-M, 22. **b** and **c**: Representative electropherograms of wild-type and mutated SNaPshot® reactions of *BRCA1* (B) and *BRCA2* (C). The nucleotides added by primer extension are shown in blue (G), black (C), red (T) and green (A);*, extension performed with an antisense primer

After minor adjustments of primer length for avoiding some peak overlapping, we were able to simultaneously detect all 8 single mutated alleles by a single PCR followed by two multiplex SNaPshot® reactions. Repeated experiments carried out on several available DNA samples gave clear cut and reproducible results. Examples of the multiplex amplified products and of the mutated and wild type patterns are illustrated in Fig. 5.

The validity and usefulness of this SNaPshot® assay for our *BRCA* pre-screening was evaluated by assaying DNA samples of patients enrolled for mutation testing, in parallel with standard screening. Among the cases with North-East Italian geographic origin, 70 probands were selected based on a *BRCA*pro value > 0.1 and/or a tumor onset before age 60 years. Twelve new probands were identified in advance as carriers of one of the common mutations, specifically one case with *BRCA2* c.8878C > T, 2 cases each with *BRCA1* c.116G > A, c.1687C > T, c.5266dupC and *BRCA2* c.7806-2A > G, 3 cases with *BRCA1*c.181T > G. All mutations were then confirmed by direct sequencing of the multiplex PCR and also of an independent single amplified product. *BRCA1* c.676delT and *BRCA2* c.5682C > G were not present in this small series of families.

With DHPLC/Sanger Sequencing/MLPA standard screening, other less common mutations were detected in only six additional families (details not reported), thus confirming an high recurrence of the founder mutations also in this subset of patient population (12/18).

## Discussion

More than a half of the tested breast/ovarian cancer patients originating from FVG and neighboring geographic areas of Veneto (Italy) and Istria (Slovenia and Croatia) were found to be carrier of one of the 8 *BRCA1* or *BRCA2* recurrent mutations. Mutations may be observed repeatedly across unrelated individuals either because the mutation arises multiple times *de novo* at hot spot DNA sites, or because it occurred once in an ancestor who then transmitted it to the progeny. To demonstrate the hypothesis of a founder origin, we explored a total of 62 families previously identified to be carriers of the 5 *BRCA1* and 3 *BRCA2* prevalent mutations in our region and surrounding territories.

The most recurrent variant was c.7806-2A > G, an intronic mutation of the *BRCA2* gene previously known as IVS16-2A > G, which severely impairs the splicing of exon 16 [28] and is predicted to remove 57 aminoacids from the encoded protein (p.Ala2603_Arg2659del). In the present series of patients it has been found in 19 unrelated probands, accounting for 8,6 % of all mutated cases. In the BIC database along with our first recorded case, only Myriad Genetics appears as depositor of this mutation (four times). However, this mutation seems to be as much or more frequent in Slovenia [29, 30] and it was originally proposed as a Slovenian founder mutation [31]. In addition, it has also been reported once in an American pancreatic cancer family [32].

In the present study, we performed haplotype analysis of 8 microsatellite markers, located in a region of approximately 7 cM surrounding the *BRCA2* gene, in the 13 Italian carrier families all from the FVG region. Our results demonstrate that the c.7806-2A > G mutation derives from a common ancestor. According to the DMLE + 2.2 software, its estimated age is around 94 generations (average of three estimations), corresponding to approximately 2350 years ago.

The recurrence in both Italian and Slovenian families suggests that this mutation has originated only once in the past, although demonstration that the c.7806-2A > G chromosomes share the same haplotype has not been explored in this study. At present, it can only be hypothesized that the c.7806-2A > G could be originated in FVG and then spread to other near areas where it is now found at appreciable frequencies, or alternatively, carriers of this mutation came from a nearby region (possibly from Slovenia), thus it became frequent in FVG. It is interesting to note that 5 of the 8 presently described mutations (*BRCA1* c.116G > A, c.181T > G; c.1687C > T and c.5266dupC, other than *BRCA2* c.7806-2A > G) correspond to highly recurrent mutations also reported in Slovenia by Krajc et al. [29–31]. This is not unexpected, if we consider the geographical proximity between Slovenia and FVG region and the common historical and political heritage over the past centuries. However, the Slovenian *BRCA1* c.844_850dupTCATTAC was not frequent in our dataset, since we found it only twice, in a North-East Italian family and in one unrelated proband from Poland.

We then chose to investigate *BRCA1* c.676delT, which accounted for almost 5 % of all mutated cases of our dataset and was not included in the Slovenian recurrent mutation list [30]. Accordingly, we found it 10 times, only in patients from the FVG area, spread in the four regional provinces. This mutation, previously defined as c.795delT in the BIC database, causes frameshift and is predicted to produce a truncated protein (p.Cys226Valfs*8). Haplotype analysis of the 9 Italian families with 7 informative microsatellite markers, located in a region of approximately 8.8 cM surrounding the *BRCA1* gene, demonstrated that also the c.676delT mutation derives from a common ancestor. Its age was estimated as an average of 86 generations, corresponding to approximately 2150 years ago. Interestingly, this mutation was recorded 16 times in the BIC database and it has been recently reported at low frequency in Austria (<2 %), which is another Nation bordering on FVG [33].

Unlike other European studies that reported a more homogeneous distribution of mutations, spread to the whole national area [4], a high degree of internal heterogeneity exists in Italy, due to past isolation of ancient populations, genetic drift and different admixture events [34]. As a consequence, several different *BRCA1* and *BRCA2* mutations have been reported that are confined within restricted geographic areas [9–15]. This seems to be the case also for some of the recurrent mutations discussed here, especially *BRCA2* c.7806-2A > G and *BRCA1* c.676delT, but also *BRCA1* c.116G > A. This latter is a missense mutation substituting a Cystein in the ring finger domain of BRCA1 protein (p.Cys39Tyr). Although it has not yet been classified by BIC and it is still not listed in the LOVD-IARC database [35], our segregation data strongly point out in favour of an important clinical role in conferring breast and ovarian cancer risk. In our series it appears frequent, but not limited to Italy, because 8 out of the 11 patients with this mutation were Italians from the borderlands of FVG and the other 3 came from Istria. Accordingly, *BRCA1* c.116G > A is also described as recurrent in the neighboring western part of Slovenia [30], but rarely reported by others.

On the basis of literature data and databases of *BRCA* variants, the remaining mutations are shared with other Italian regions, such as the nonsense *BRCA2* c.8878C > T, or have a broader diffusion among Caucasians in Europe, such as the *BRCA1* c.181T > G and c.1687C > T and *BRCA2* c.5682C > G [30, 36–38], or worldwide, such as *BRCA1* c.5266dupC [8, 39].

With regard to the *BRCA1* c.5266dupC, we have found it 15 times. However, in this series of cases only a minority had a North-East Italian origin, being 9 of the identified carriers from other non-neighbouring Italian regions. Despite this, by microsatellite analyses we have obtained evidence of significant haplotype sharing among carriers (alleles 151–247 at *loci* D17S855 and D17S1328). c.5266dupC, formerly known as 5382insC, was originally described as a founder mutation in the AJ population [4]. However, an extended haplotype study on 14 different population groups demonstrated that all mutation carriers share a common haplotype that arose 1800 years ago from a single Scandinavian or Russian founder individual [40]. Thus, it was a common European mutation long before becoming an AJ founder mutation.

Another useful observation we can gather from our study concerns the clinical phenotype associated to *BRCA2* c.7806-2A > G. We had already reported its geographical recurrence in our previous study in which we also underlined its possible role in predisposition to male breast cancer [41]. In *BRCA2* mutation carriers the cumulative risk of male breast cancer at age 70 years has been estimated 6.8 % [42], but evidences for a correlation between the location of the mutation within *BRCA2* gene and risk of male breast cancer are still lacking [43]. The data presented here suggest an association for c.7806-2A > G, but further studies are necessary for providing a more precise estimate for a male mutation carrier of the likelihood of developing breast cancer.

The identification of mutations is efficient and cost-effective when testing can be limited to a number of common founder mutations within a defined ethnic and/or geographical group. However, in our patient population, genetic testing of high risk families cannot be restricted to a small number of mutations, since about half were unique or poorly recurrent. Although sequencing of the two genes in their entirety is still necessary, it may be advantageous adopting the SNaPshot® assay we have developed for rapidly pre-screening the 8-mutation panel. Customized *BRCA1* and *BRCA2* SNaPshot® tests have been previously implemented also by other groups for assaying their own most common mutations [44, 45].

Target re-sequencing on a Next Generation Sequencing instrument is now available in our laboratory to overcome the increasing demand of rapid testing, often for orienting surgical or therapeutic decisions. However, the proposed genotyping strategy is still an useful option for less equipped laboratories and could also be adopted as a cost-effective approach for testing larger populations of patients in North-East of Italy.

## Conclusions

Five *BRCA1* and 3 *BRCA2* recurrent mutations account for more than half of the patients with proven hereditary breast/ovarian cancer originating from FVG and neighboring geographic areas. Proofs of common ancestry has been obtained for all eight mutations, also providing evidence that *BRCA1* c.676delT and *BRCA2* c.7806-2A > G arose around 90 generations ago. Rapid genotyping of these highly recurrent mutations could be offered to a larger number of breast and/or ovarian cancer patients with North-East Italian origin, irrespective of the established diagnostic criteria for hereditary tumors.

## Abbreviations

AJ: Ashkenazi Jews
FVG: Friuli Venezia Giulia
DHPLC: Denaturing High Performance Liquid Cromatography
MLPA: Multiplex-Ligation Dependent Probe Amplification
SAP: Shrimp Alkaline Phosphatase
